# METTL14 promotes IL‐6‐induced viability, glycolysis and inflammation in HaCaT cells via the m6A modification of TRIM27

**DOI:** 10.1111/jcmm.18085

**Published:** 2023-12-25

**Authors:** Yiran Chen, Yanwei Xiang, Xiao Miao, Le Kuai, Xiaojie Ding, Tian Ma, Bin Li, Bin Fan

**Affiliations:** ^1^ Department of Dermatology, Yueyang Hospital of Integrated Traditional Chinese and Western Medicine Shanghai University of Traditional Chinese Medicine Shanghai China; ^2^ Engineering Research Center of Traditional Chinese Medicine Intelligent Rehabilitation Ministry of Education Shanghai China; ^3^ School of Rehabilitation Science Shanghai University of Traditional Chinese Medicine Shanghai China; ^4^ Innovation Research Institute of Traditional Chinese Medicine Shanghai University of Traditional Chinese Medicine Shanghai China; ^5^ Shanghai Skin Disease Hospital, School of Medicine Tongji University Shanghai China

**Keywords:** IL‐6, m^6^A methylation, METTL14, psoriasis, TRIM27

## Abstract

Interleukin‐6 (IL‐6) is a cytokine generated by healthy constituents of the skin, but is also up‐regulated by a wide range of skin lesions and inflammatory conditions to trigger cytopathy of skin cells. TRIM27 was identified to contribute to the functional effects of IL‐6 on skin cells. However, the underlying mechanism was not clear. Lentivirus infection was used for gene overexpression or silencing. RT‐PCR and Western blot were used to respectively assess mRNA and protein levels. Cell viability was assessed by CCK‐8 assay. Extracellular flux analysis was used to assess the levels of oxygen consumption rate and extracellular acidification rate. Mouse back skin was treated with imiquimod to produce psoriasis‐like inflammation in vivo. Histological assessment and immunohistochemistry staining were respectively applied to analyse lesioned mouse and human skin samples. IL‐6‐induced increased viability, glycolysis and inflammation in keratinocytes was inhibited both by a chemical methylation inhibitor and by METTL14 knockdown. Further investigation found that METTL14 induces m6A methylation of TRIM27, which is recognized by a m6A reader, IGF2BP2. Elevation of TRIM27 level and activation of IL‐6/STAT3 signalling pathway were found in an in vivo psoriasis‐like inflammation model, whereas inhibition m6A methylation strongly alleviated the inflammation. Finally, METTL14, TRIM27, STAT3, p‐STAT3 and IL‐6 expressions were all found to be increased in clinical skin samples of psoriatic patients. Our results unravelled METTL14/TRIM27/IGF2BP2 signalling axis in keratinocyte cytopathy, which plays a critical role in facilitating the activation of IL‐6/STAT3 signalling pathway. Our findings should provide inspirations for the design of new therapeutics for skin inflammatory diseases including psoriasis.

## 1 INTRODUCTION

Psoriasis is a chronic inflammatory skin disease involving the hyperproliferation of keratinocytes, and cellular inflammatory infiltration.^1^ The pathogenesis of it is not completely clear, but it is known that many immune signalling pathways perform an important role in the occurrence and development of psoriasis.^2^, ^3^ During inflammation, cellular glucose uptake and glycolysis are upregulated to meet an increased energy demand. For example, keratinocyte glycolysis is essential for progression of psoriasis.^4^ Therefore, understanding the regulation of glucose metabolism in keratinocytes is of importance.

Interleukin‐6 (IL‐6) is a cytokine generated and secreted by healthy constituents of the skin. In the meanwhile, it is up‐regulated by and closely mediates the development of a wide range of skin lesions such as psoriasis^5^, ^6^ and inflammatory responses,^7^, ^8^ and it also plays an indispensable role in cutaneous wound healing.^9^, ^10^ As a well‐known pro‐inflammatory cytokine in multiple cell and tissue systems,^11^ IL‐6 has served as a popular target for the design and development of anti‐inflammatory agents,^12^ including ones for treating chronic skin diseases.^13^ However, it has been widely accepted for the last decade that IL‐6 also has anti‐inflammatory properties.^14^, ^15^, ^16^ recent research has shown that IL‐6 secreting MSC accelerates diabetes reversal through the upregulation of IL‐17A and TGF‐β in the skin.^17^ Moreover, IL‐6 induces hyperproliferation and inflammation of cells, which is at least in part via the IL‐6/STAT3 (signal transducer and activator of transcription) signalling pathway,^18^ a crucial signalling pathway identified in many diseases.^19^ Importantly, in human keratinocytes, tripartite motif 27 (TRIM27) was found to be involved in the IL‐6/STAT3 signalling pathway by binding to PIAS3 and facilitating its ubiquitination.^18^ However, exactly how TRIM27 is regulated in psoriasis has never been discussed.

Interestingly, previous works have shown that psoriasis is associated with transcriptome‐wide m^6^A methylation in diseased skin tissues,^20^ which implied a strong contribution of m^6^A modification writers to IL‐6‐induced keratinocyte cytopathy. Indeed, an association of m^6^A modification writers with IL‐6 signalling were already noted in many other diseases. In the pathological condition of cholangiocarcinoma, m^6^A modification writers METTL3, METTL14, and Wilms' tumour 1‐associating protein were found to be up‐regulated by IL‐6 in the diseased tissues.^21^ In atherosclerosis, METTL14 was again found to mediate the inflammatory response of macrophages via the regulation of IL‐6.^22^ Considering that these above results all underscore a linkage between METTL14 and IL‐6, in this work we explored the involvement of METTL14 in IL‐6‐induced keratinocyte cytopathy with an emphasize on psoriasis. Our results showed that METTL14 plays a facilitating role in IL‐6‐induced viability, glycolysis and inflammation in HaCaT cells, a human immortalized keratinocyte, via the m^6^A modification of TRIM27. Our work identified a critical linking point between the IL‐6/STAT3 signalling pathway and m^6^A methylation, and unravelled METTL14 as an important player in IL‐6‐induced keratinocyte cytopathy.

## 2 MATERIALS AND METHODS

### 2.1 Cell culture and treatment

HaCaT cells (BeNa Culture Collection, Suzhou, China) were cultured under 37°C and 5% CO_2_ using DMEM medium with 10% foetal bovine serum (FBS) and 1% streptomycin–penicillin solution (Gibco, USA). To generate an in vitro model of keratinocyte that replicated the features of psoriatic skin and the effect of chemical suppression of m6A methylation by 3‐deazaadenosine (DAA), HaCaT cells were treated with 50 μM DAA (Cayman Chemical, USA) for 2 h prior to 10 ng/mL IL‐6 (Sigma, USA) for 48 h.^23^, ^24^

### 2.2 Cell transfection

siRNAs and shRNAs were used for gene silencing. Two IGF2BP2 siRNAs (siIGF2BP2–1: 5′‐GCAACAAGAGAAGAAGCAATT‐3′, siIGF2BP2–2: 5′‐GGCAGAUGAGACCAAACUATT‐3′) were constructed by Genepharm Technologies (Shanghai, China). TRIM27‐specific shRNA (5′‐CCCAGTTCTCTTGCAACAT‐3′) and METTL14‐specific shRNAs (shMETTL14‐1: 5′‐CGGAAGAAAGGTTGGATAA‐3′, shMETTL14‐2: 5′‐GGATGAACTAGAAATGCAA‐3′ and shMETTL14‐3: 5′‐GGAAGAGTGTGTTTACGAA‐3′) were inserted into the pLKO.1 vector (Addgene). METTL14 overexpression lentiviral vector was constructed by fusing the coding sequence of METTL14 into pLVX‐Puro (Clontech Laboratories, Inc., Mountain View, CA, USA). The vectors were transfected into 293 T cells using Lipofectamine 2000 reagent (Invitrogen) to produce viruses, which were then used for cell infection. The scramble siRNA (siNC), scramble shRNA (shNC) and blank pLVX‐Puro (Vector) were used as negative controls.

### 2.3 CCK‐8 assay used for assessing cell viability

The viability of HaCaT cells was analysed by the CCK‐8 method using the cell counting kit‐8 assay kit obtained from Dojindo Molecular Technologies (Japan) as the instruction of the reagent. Briefly, HaCaT cells were inoculated into 96‐well plates. At 6, 12, 24 and 48 h after treatment, each well was added with 10 μL of CCK‐8 solution and incubated for 1 h. Absorbance values were measure at 450 nm.

### 2.4 Extracellular flux analysis

The level of oxygen consumption rate (OCR) and extracellular acidification rate (ECAR) was estimated using Seahorse XF24 Extracellular Flux Analyser.^25^ Briefly, cells (1 × 10^4^/well) were incubated under 37°C and 5% CO_2_ for 24 h. Around 1 h before testing, CO_2_ was disconnected and culture medium was replaced by XF Base Medium (Agilent Technologies). Then 1 μM of oligomycin, 1.5 μM of carbonyl cyanide p‐trifluoromethoxyphenylhydrazone and a mixture of antimycin A (0.5 μM) and rotenone (0.5 μM) were respectively added into ‘A’, ‘B’ and ‘C’ well of the Seahorse gauging plate. The cellular OCR was then monitored. For measurement of ECAR, the cells were treated sequentially with 1 μM of glucose, 1 μM of oligomycin and 0.5 μM of 2‐DG.

### 2.5 Measurement of lactate

Cells were cultured overnight at 37°C, 5% CO_2_, and then the medium was replaced by fresh DMEM, and the cells were cultured for another 24 h. Cell supernatant was collected after centrifugation, and the lactate release in the supernatant was determined using Lactic Acid assay kit (Nanjing Jiancheng Bioengineering Institute, China).

### 2.6 ELISA

The levels of IL‐1β, IL‐6 and IL‐8 in cell supernatants were analysed respectively using Human IL‐1 beta Quantikine ELISA Kit (R&D Systems, Minneapolis, MN, USA), Human IL‐6 ELISA Kit (Abcam, ab178013) and IL‐8 Human ELISA Kit (Abcam, ab214030).

### 2.7 Quantitative RT‐PCR

Total RNA was extracted using a TRIzol reagent (Life Technologies, Inc., Waltham, MA, USA). cDNA was synthesized using a PrimeScript kit (Takara Biotechnology, Dalian, China). Quantitative RT‐PCR was performed in an ABI 9700 real‐time PCR system (Applied Biosystem). The primers were shown as follows: METTL14: F‐5’‐CTGGGAATGAAGTCAGGATAG‐3′, R‐5’‐CCAGGGTATGGAACGTAATAG‐3′; TRIM27: F‐5’‐TGCCATCACCCAGTTCTC‐3′, R‐5’‐AGCCCTGCTCAATGTGTC‐3′; IGF2BP2: F‐5’‐CGGGAGCAAACCAAAGACC‐3′, R‐5’‐GCAAACCTGGCTGACCTTC‐3′; GAPDH: F‐5’‐AATCCCATCACCATCTTC ‐3′, R‐5’‐AGGCTGTTGTCATACTTC‐3′.

### 2.8 Western blot analysis

Protein lysates were prepared using RIPA lysis buffer containing protease inhibitor cocktail (Sigma, St. Louis, MO, USA). After being separated by SDS‐PAGE, proteins were transferred onto nitrocellulose membranes (Millipore, Bedford, USA), blocked with 5% skim milk, and incubated with primary antibodies against METTL14 (Abcam; ab220030; 1:500), TRIM27 (Abcam; ab78393; 1:1000), STAT3 (Abcam; ab68153; 1:1000), p‐STAT3 (Abcam; ab267373; 1:1000), IGF2BP2 (Abcam; ab129071; 1:1000), and β‐actin (Cell Signalling Technology; 4970 s; 1:1000), followed by incubation with HRP‐conjugated secondary antibodies (Beyotime, Shanghai, China; A0208, A0216; 1:1000). Signals were detected using enhanced chemiluminescence system (Bio‐Rad, Richmond, CA, USA).

### 2.9 m^6^A content analysis

Trizol reagent was used to extract the total RNA. Poly (A)^+^ RNA was purified using GenElute™ mRNA Miniprep Kit (Sigma, Louis, MO, USA; MRN10). The m^6^A content was assayed using the m^6^A RNA Methylation Assay Kit (Abcam, ab185912).

### 2.10 RNA immunoprecipitation (RIP) assays

RIP assays were carried out using the Magna RIP RNA‐Binding Protein Immunoprecipitation kit (Millipore, Billerica, MA, USA; 17–701). RNA‐protein complexes were conjugated with anti‐m6A (Abcam, ab208577), anti‐IGF2BP1 (ab184305), anti‐IGF2BP2 (ab128175), anti‐IGF2BP3 (ab177477), or anti‐IgG antibody (ab172730) at 4°C for 1 h. Then cells were incubated with garose beads and 50 μL of protein A/G for another 60 min at 4°C. The precipitated beads were washed with RIP‐wash buffer for 10 min at 4°C and then RIP‐lysis buffer for 5 min at 4°C. Cells were incubated at 65°C for 2 h with 200 mM NaCl and 20 μg proteinase K to release the RNA in the immunoprecipitated complex and the RNA in the previously saved input fraction. The co‐precipitated RNAs were purified using phenol:chloroform:isoamyl alcohol and subjected to quantitative RT‐PCR.

### 2.11 Reporter gene assays

The TRIM27 3′UTR sequence was cloned into the pGL3 vector (Promega, Madison, WI, USA). HaCaT cells were transduced with METTL14 shRNA vector (co‐transfected with either the pGL3‐TRIM27 3′UTR luciferase reporter plasmid or the internal reference plasmid pRL‐TK vector. Then a dual‐luciferase assay was conducted.

### 2.12 mRNA stability measurements

HaCaT cells were treated with 0.2 mM actinomycin D (GlpBio, Montclair, CA, USA; GC16866) and then collected to extract the total RNA. cDNA synthesis by reverse transcriptase was subsequently performed using an oligo(dT) primer. The mRNA levels were determined by quantitative RT‐PCR.

### 2.13 Mice and treatments

All animal experiments were approved by the Ethics Committee of Yueyang Hospital for the use of animals and conducted in accordance with the National Institutes of Health Laboratory Animal Care and Use Guidelines. Eighteen male and female 8‐week‐old BALB/c mice were purchased from CEBIO (ICB, UFMG, Belo Horizonte, MG, Brazil) and randomly divided into three groups: control group; IMQ group; and IMQ + DAA group (three male and three female mice in each group). A topical dose of 62.5 mg IMQ cream (Sichuan Mingxin Pharmaceutical Co., Ltd., Sichuan, China) was applied to the shaved back region of mice for 6 days continuously, while a vehicle cream (Vaseline Lanette cream; Fagron) was used to treat the control mice. From day 7 to 12, mice were intraperitoneal injection of 800 mg/kg DAA. The severity of each lesion was graded and monitored using an improved human scoring system, the psoriasis area severity index (PASI), which includes the area of the skin lesions, erythema, scaling and thickening. The PASI scores are 0 (none); 1 (light); 2 (moderate); 3 (severe); and 4 (extremely severe).^26^

### 2.14 Histological assessment

Skin fragments from back region of mice were fixed within 10% formalin, dehydrated and embedded into paraffin, which were then sliced into sections (thickness: 4 μm) and stained with haematoxylin and eosin (H&E). Images were captured at 200× magnification using a microscope (Olympus BX51).

### 2.15 Clinical samples

Thirty untreated patients with psoriasis were recruited from the Yueyang Hospital of Integrated Traditional Chinese and Western Medicine with written informed consent, and skin tissues were collected from those with plaque psoriasis covering at least 10% of their total body surface for at least 6 months. Skin tissues obtained from 10 healthy volunteers were used as control. All procedures performed in studies involving human participants were in accordance with the standards upheld by the Ethics Committee of Yueyang Hospital and with those of the 1964 Helsinki Declaration and its later amendments for ethical research involving human subjects (Approval No. YYLAC‐ 2019‐014‐4). Written informed consent was obtained from all the patients.

### 2.16 Immunohistochemistry (IHC) staining

Protein analysis was performed by IHC staining of lesioned skin tissues using antibodies against METTL14 (Abcam; ab220030; 1:1000), TRIM27 (Abcam; ab78393; 1:1000), p‐STAT3 (Cell Signalling Technology; 9145; 1:100) and IL‐6 (Abcam; ab9324; 1:100). All sections were viewed under a light microscope (Olympus BX41; Olympus Corporation) and analysed using Cell D1 Image analysis software (Olympus Corporation). The H‐score system was used to assess immunoreactivity by two investigators based on the percentage of positively stained cells (graded on a scale of 0–4: 0, <5%; 1, 5%–25%; 2, 25%–50%; 3, 50%–75%; 4, >75%) and the intensity of staining (graded on a scale of 0–3: 0, negative; 1, weak; 2, moderate; 3, strong), which ranged from 0 to 12.

### 2.17 Statistical analysis

All experiments were conducted in triplicates and data were expressed as mean ± SD. All statistical analyses were done with GraphPad Prism 8.4.2 (GraphPad Software, San Diego, CA, USA). Comparisons of two groups were done with Student's *t*‐test, and comparisons of three or more groups were done with one‐way ANOVA. A *p*‐value of <0.05 indicated statistical significance.

## 3 RESULTS

### 3.1 DAA inhibits IL‐6‐induced viability, glycolysis and inflammation in keratinocytes

**FIGURE 1:**
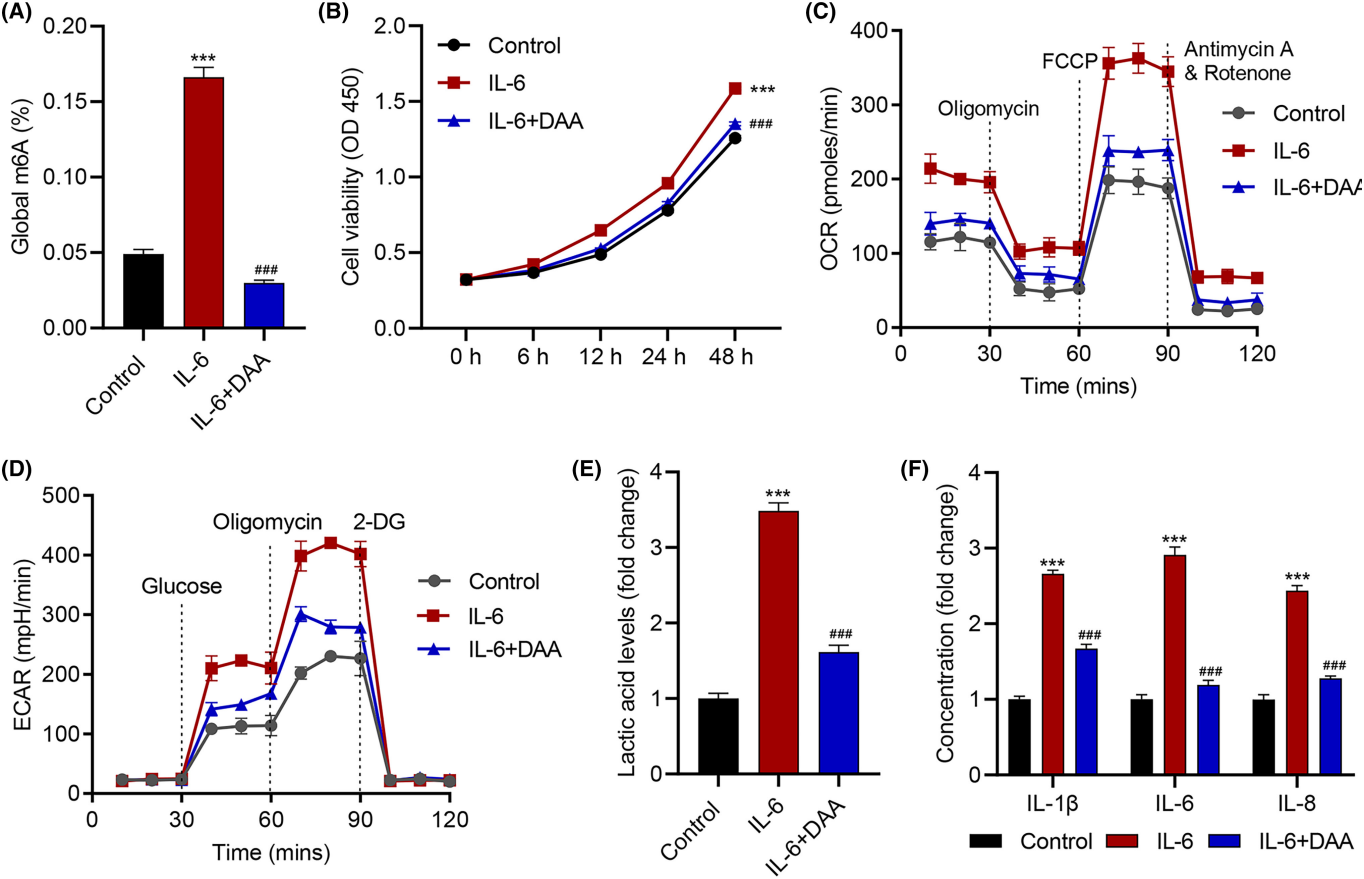
DAA inhibits IL‐6‐induced HaCaT cell viability, glycolysis and inflammation. (A) global m6A levels, (B) cell viability, (C) OCR, (D) ECAR, (E) lactic acid, (F) IL‐1β, IL‐6, and IL‐8 release in HaCaT cells treated with 50 μM DAA for 2 h prior to 10 ng/mL IL‐6 for 48 h. Data are presented as mean ± SD from three independent experiments. ***p < 0.001 compared with control. ^###^ *p* < 0.001 compared with IL‐6.

HaCaT cell line was used as an in vitro model of keratinocytes, and soluble IL‐6 was added into the cell suspension to stimulate the cells. To examine the relation of m^6^A methylation with IL‐6 signalling, we extracted Poly (A)^+^ RNA from the cells and measured their global m^6^A level. The results showed a significant increase of m^6^A level after IL‐6 treatment (*p* < 0.001; Figure 1A). To check the effects of IL‐6 in inducing cell viability, CCK‐8 assay was used for measuring cell viability after IL‐6 treatment. Agreeing with previous works,^18^, ^27^ cells treated with IL‐6 gradually manifested higher viability than those without IL‐6 treatment at 48 h (*p* < 0.001; Figure 1B). Higher proliferative activities require quicker energy metabolism, that is, faster consumption of oxygen and energy, a signature behaviour of cancer cells.^28^ We therefore further measured how IL‐6 affected the OCR and ECAR, an indicator of the rate of glycolysis.^29^, ^30^ As the results showed, IL‐6 substantially increased both OCR and ECAR of HaCaT cells (Figure 1C, D). Agreeing with this, the level of lactic acid, an end product of glycolysis,^31^ in the cells were also greatly elevated after IL‐6 treatment (*p* < 0.001; Figure 1E). On the other hand, using enzyme‐linked immunoassay (ELISA), the proinflammatory effects of IL‐6 in HaCaT cells was clearly reflected by an increase in IL‐1β, IL‐6 and IL‐8 release (*p* < 0.001; Figure 1F).^32^

In order to figure out the relation of m^6^A methylation with IL‐6‐induced keratinocyte cytopathy, DAA, a commonly used methylation inhibitor that suppresses SAH hydrolase and disrupts insertion of m^6^A into mRNA substrates,^33^ was used to treat the cells in parallel with IL‐6. Indeed, the addition of DAA decreased the m^6^A level that was up‐regulated by IL‐6, confirming that it is indeed an effective m^6^A methylation inhibitor (*p* < 0.001; Figure 1A). Interestingly, DAA also significantly decreased IL‐6‐induced elevation of cell viability (*p* < 0.001; Figure 1B), OCR and ECAR (Figure 1C, D), lactic acid level (*p* < 0.001; Figure 1E) as well as the levels of IL‐1β, IL‐6, and IL‐8 release (*p* < 0.001; Figure 1F). These results strongly suggested a contributing role of m^6^A methylation in IL‐6‐induced viability, glycolysis and inflammation in keratinocytes.

### 3.2 METTL14 knockdown inhibits IL‐6‐induced viability, glycolysis and inflammation in keratinocytes

**FIGURE 2:**
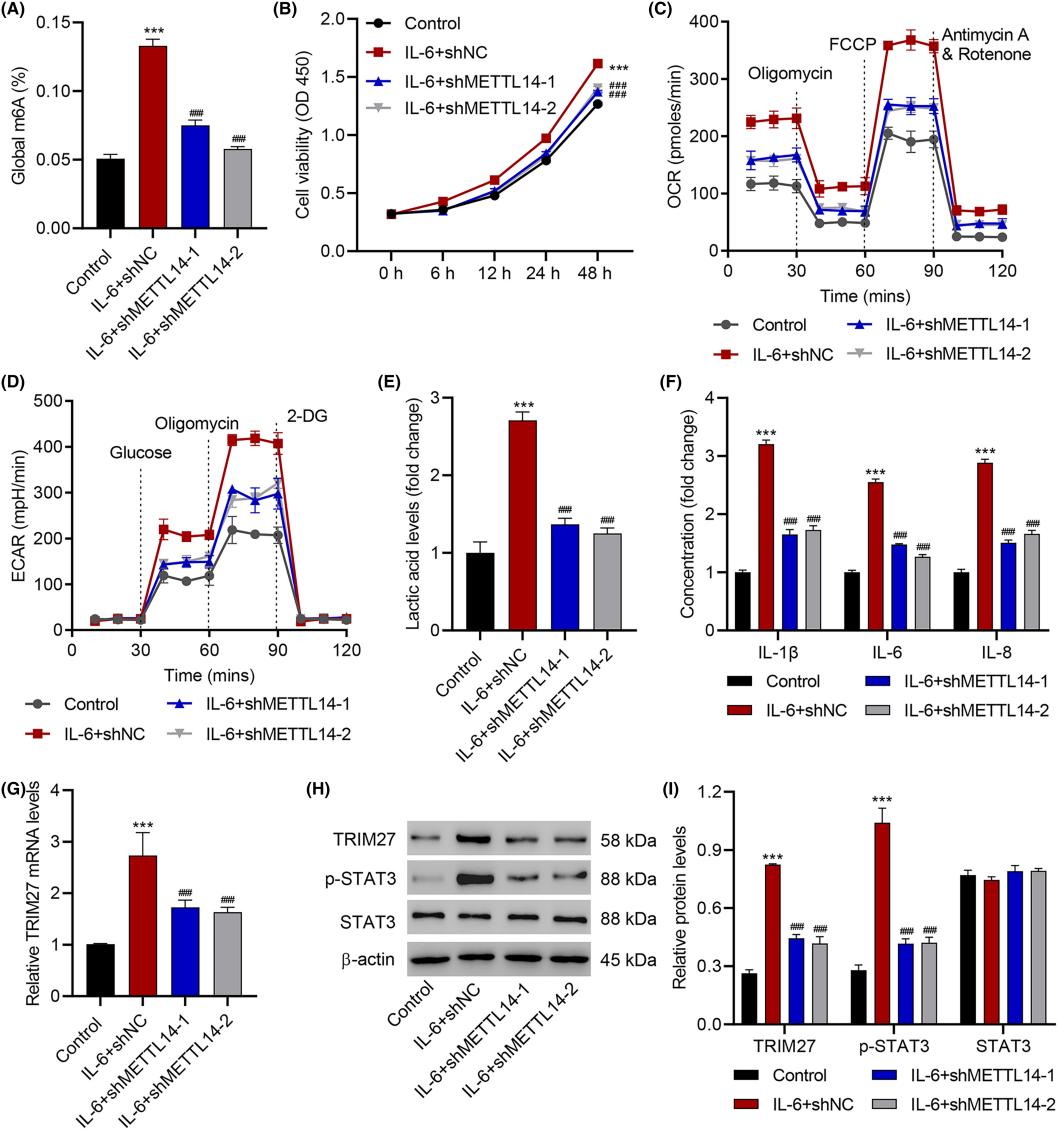
METTL14 knockdown inhibits IL‐6‐induced HaCaT cell viability, glycolysis and inflammation. (A) global m6A levels, (B) cell viability, (C) OCR, (D) ECAR, (E) lactic acid, (F) IL‐1β, IL‐6, IL‐8 release, (G) TRIM27 mRNA level and (H, I) expression of TRIM27, STAT3 and p‐STAT3 in HaCaT cells transduced with METTL14 shRNA vector prior to 10 ng/mL IL‐6 treatment for 48 h. Data are presented as mean ± SD from three independent experiments. ***P < 0.001 compared with control. ^###^ *p* < 0.001 compared with IL‐6 + shNC.

We next examined the involvement of METTL14 in IL‐6‐induced keratinocyte cytopathy by knocking down METTL14 in HaCaT cells with the infection of METTL14‐specific shRNAs. Among the three shRNAs tested, shMETTL14‐1, −2 and −3, all three of them effectively reduced the mRNA level of METTL14. However, shMETTL14‐1 and − 2 clearly had a better effect in inhibiting the METTL14 protein expression (*p* < 0.001; Figure S1A). Therefore, for further experiments, shMETTL14‐1 and − 2 were used for cell infection to achieve METTL14 knockdown. Importantly, METTL14 knockdown greatly reduced global m^6^A level in the cells after IL‐6 treatment (*p* < 0.001; Figure 2A). Furthermore, METTL14 knockdown also significantly decreased cell viability (*p* < 0.001; Figure 2B), OCR (Figure 2C), ECAR (Figure 2D) and lactic acid (*p* < 0.001; Figure 2E) and IL‐1β, IL‐6, and IL‐8 levels (*p* < 0.001; Figure 2F) in the HaCaT cells after IL‐6 treatment. These results suggested that METTL14 is critical to IL‐6‐induced viability, glycolysis and inflammation in keratinocytes, and it is also involved in IL‐6‐induced m^6^A methylation.

TRIM27 was found in previous literature to be expressed upon IL‐6 stimulation and promote STAT3 activation.^18^ We hereby hypothesized that METTL14 can regulate the m^6^A methylation of TRIM27, thereby facilitating the signalling of IL‐6 towards STAT3 activation. To test this hypothesis, we first checked the mRNA and protein levels of TRIM27 after IL‐6 stimulation, with and without METTL14 knockdown. As the results showed, METTL14 knockdown substantially reduced the transcription and expression of TRIM27 (*p* < 0.001; Figure 2G–I). More importantly, as revealed by Western blot, while the overall expression of STAT3 remained unchanged, the level of phosphorylated STAT3 (p‐STAT3), that is, the active form of STAT3, became much higher after IL‐6 stimulation but was brought down by METTL14 knockdown (*p* < 0.001; Figure 2H,I).

### 3.3 TRIM27 knockdown inhibits METTL14‐induced viability, glycolysis and inflammation in keratinocytes

**FIGURE 3:**
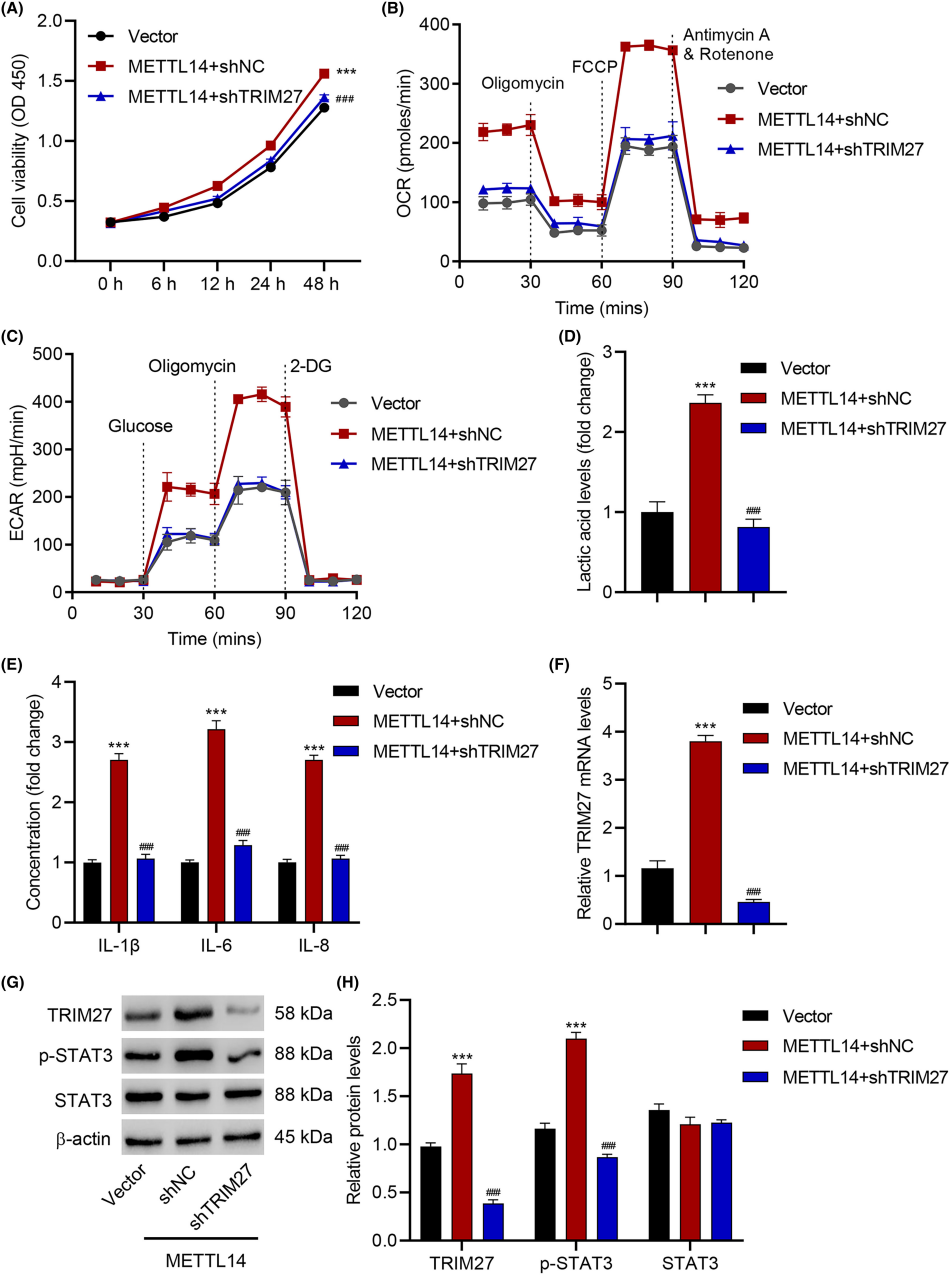
TRIM27 knockdown inhibits METTL14‐induced HaCaT cell viability, glycolysis and inflammation. (A) Cell viability, (B) OCR, (C) ECAR, (D) lactic acid, (E) IL‐1β, IL‐6, IL‐8 release, (F) TRIM27 mRNA level and (G, H) expression of TRIM27, STAT3 and p‐STAT3 in HaCaT cells transduced with METTL14 expression vector and with or without TRIM27 silencing for 48 h. Data are presented as mean ± SD from three independent experiments. ***p < 0.001 compared with vector. ^###^ *p* < 0.001 compared with METTL14 + shNC.

The above results confirmed that METTL14 controls the expression of TRIM27, and that it regulates IL‐6‐induced viability, glycolysis and inflammation in keratinocytes via the IL‐6/STAT3 pathway. However, it does not prove that the METTL14/TRIM27 signalling axis directly contributes to the keratinocyte cytopathy. To test this hypothesis, we silenced TRIM27 using the TRIM27‐specific shRNA (shTRIM27) and checked how it affects the functional effects of METTL14 over‐expression (using a METTL14 overexpression lentiviral vector; *p* < 0.001; Figure S1B). METTL14 overexpression manifested higher viability, OCR, ECAR, and lactic acid, IL‐1β, IL‐6, and IL‐8 levels, while TRIM27 silencing significantly attenuated all the above effects back to the background levels (*p* < 0.001; Figure 3A–E). Furthermore, TRIM27 silencing also reduced the intensified STAT3 activation triggered by METTL14 overexpression, reflected by the lowered p‐STAT3 level (*p* < 0.001; Figure 3F–H). Overall, these results indicated that METTL14 is directly upstream of TRIM27 in the IL‐6/STAT3 signalling pathway for regulating keratinocyte cytopathy.

### 3.4 METTL14 induces m6A modification of TRIM27 in IL‐6‐stimulated keratinocytes

**FIGURE 4:**
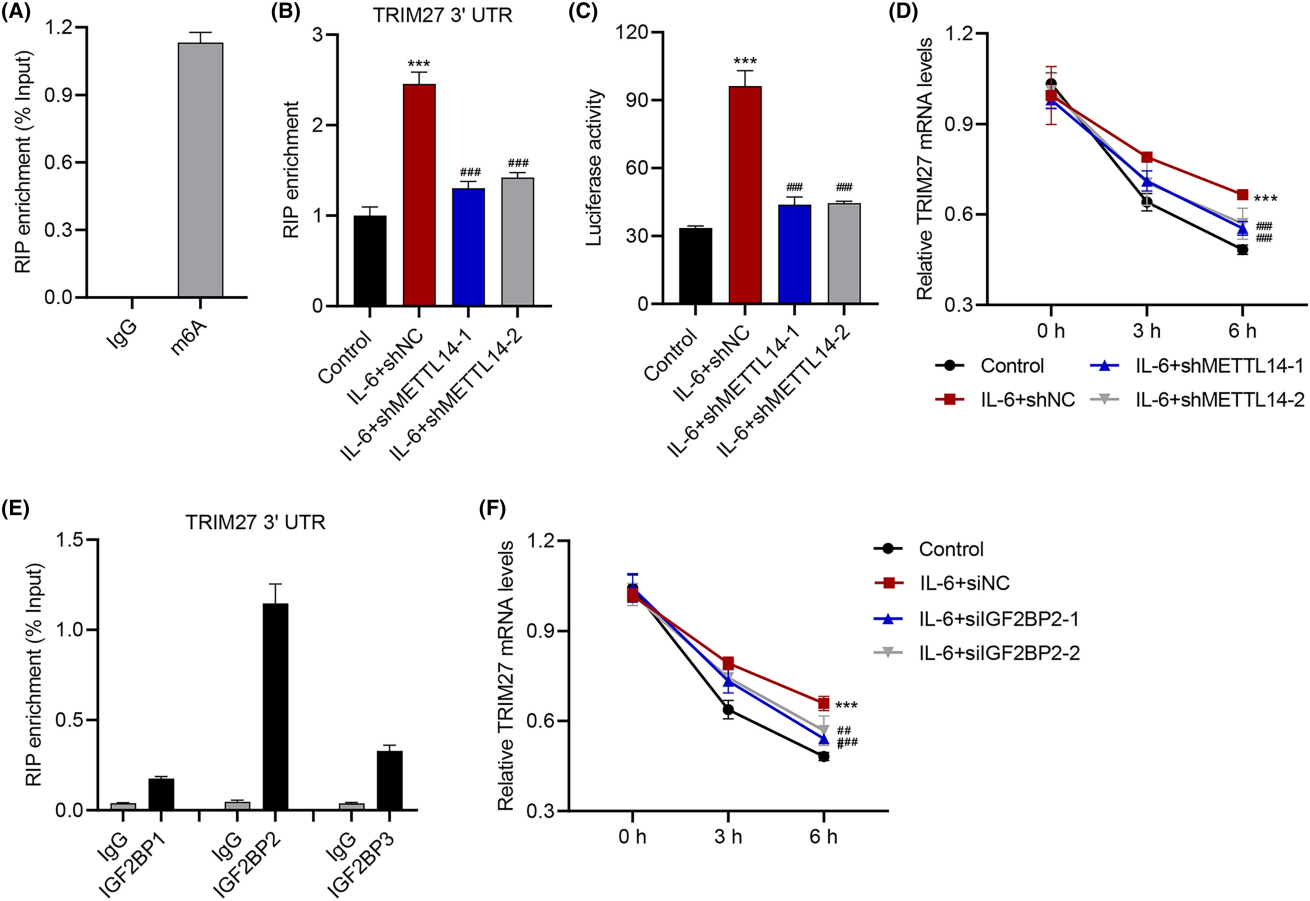
METTL14 induces m6A modification of TRIM27 in IL‐6‐stimulated HaCaT cells. (A) RIP‐PCR of TRIM27 3′UTR in HaCaT cells treated with 10 ng/mL IL‐6 immunoprecipitated by IgG or anti‐m^6^A. (B) RIP‐PCR of TRIM27 3′UTR (immunoprecipitated by anti‐m^6^A) in HaCaT cells transduced with METTL14 shRNA vector prior to 10 ng/mL IL‐6 treatment. (C) Luciferase activity of TRIM27 3′UTR in HaCaT cells transduced with METTL14 shRNA vector prior to 10 ng/mL IL‐6 treatment. (D, F) The half‐life curve of TRIM27 transcription in HaCaT cells transduced with (D) METTL14 shRNA vector or (F) transfected with IGF2BP2 siRNA, prior to 10 ng/mL IL‐6 treatment. (E) RIP‐PCR of TRIM27 3′UTR in HaCaT cells immunoprecipitated by IgG or anti‐IGF2BPs. Data are presented as mean ± SD from three independent experiments. ***p < 0.001 compared with control. ^###^ *p* < 0.001 compared with IL‐6 + shNC or IL‐6 + siNC.

The possible methylation sites in the TRIM27 mRNA sequence was predicted by Sequence‐based RNA Adenosine Methylation Site Predictor (SRAMP). To further validate that METTL14 triggers m^6^A methylation of TRIM27, RIP was used to extract TRIM27 3′UTR tagged with anti‐m^6^A antibody (Figure 4A). Notably, if the cells were pre‐infected with METTL14 shRNAs, then the m^6^A‐tagged RIP enrichment of TRIM27 3′UTR after IL‐6 stimulation became much lower, indicating reduced m^6^A methylation (*p* < 0.001; Figure 4B). Consistently, it was found in a dual‐luciferase assay that METTL14 silencing effectively reduced the luciferase activity of TRIM27 3′UTR by IL‐6 stimulation (*p* < 0.001; Figure 4C), and that the TRIM27 mRNA level also decayed faster with METTL14 silencing (*p* < 0.001; Figure 4D), suggesting lower mRNA stability.

Insulin‐like growth factor 2 mRNA binding protein (IGF2BP) is a common m^6^A reader that enhances mRNA stability and translation upon m^6^A recognition.^34^ RIP‐PCR was used to validate the interaction between TRIM27 3′UTR and IGF2BPs such as IGF2BP1, IGF2BP2 and IGF2BP3 (Figure 4E). Compared with IGF2BP1 and IGF2BP3, a higher binding ability was found between IGF2BP2 and TRIM27 3′UTR. Therefore, IGF2BP2 was used as a potential reader. To assess the involvement of IGF2BP2 in our system, two IGF2BP2 siRNAs, siIGF2BP2–1 and − 2 were used to transfect the HaCaT cells, which successfully decreased the mRNA level of IGF2BP2 to 30% or lower, and also drastically decreased IGF2BP2 protein level (*p* < 0.001; Figure S1C). Importantly, mimicking the effect of METTL14 silencing, IGF2BP2 silencing also decreased the stability of TRIM27 mRNA (*p* < 0.001; Figure 4F). Therefore, IGF2BP2 should serve as the reader of m^6^A methylation in TRIM27.

### 3.5 DAA inhibits IMQ‐induced hyperproliferation and inflammation in mice

**FIGURE 5:**
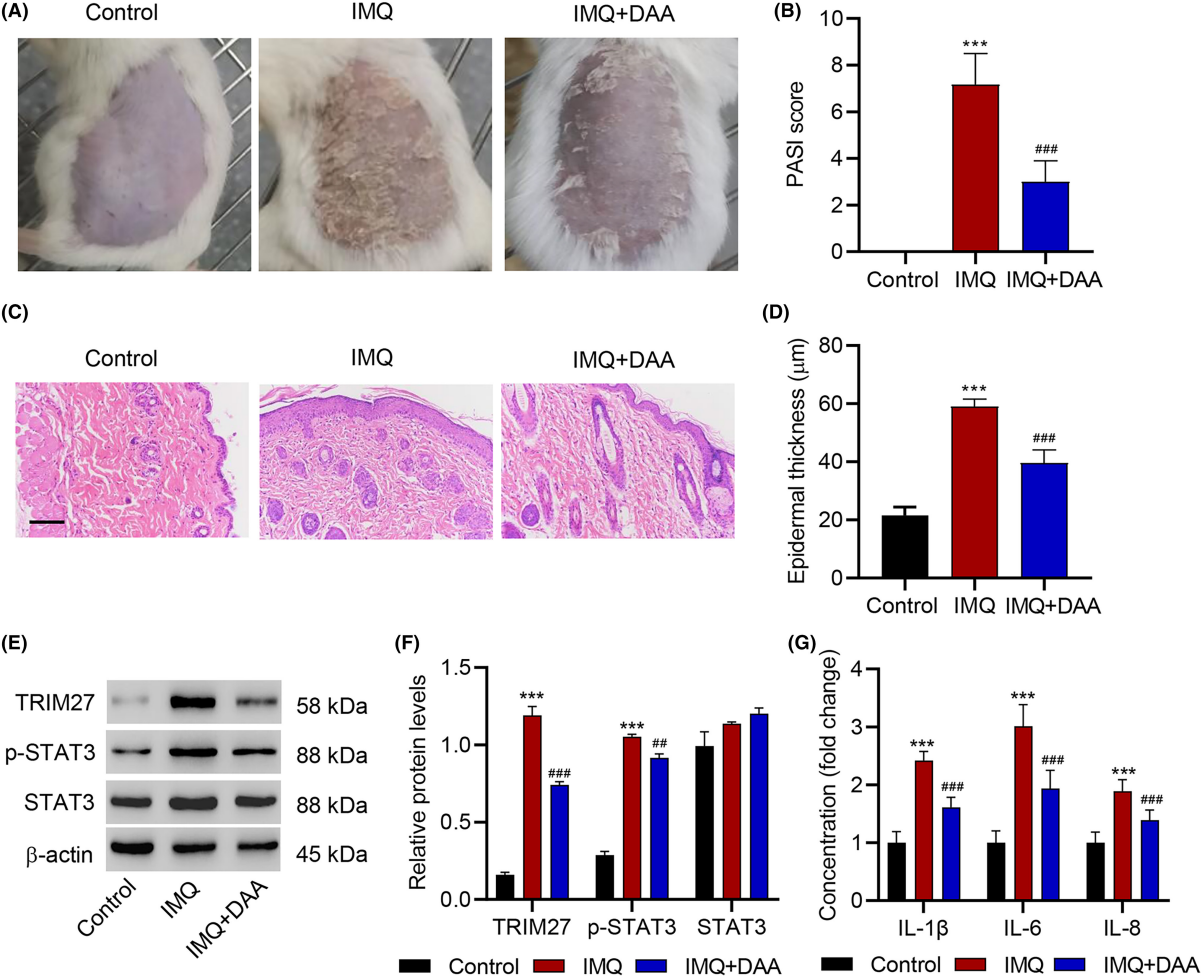
DAA inhibits IMQ‐induced hyperproliferation and inflammation in mice. Mice were treated with imiquimod (IMQ) and DAA. (A) Photographs of the backs of mice after the IMQ and DAA application. (B) Severity of psoriasis during the course of IMQ‐induced psoriasis, was evaluated by the scores on the psoriasis area and severity index (PASI) (*n* = 6). (C) H&E staining of the back skin of mice (Scale bar, 100 μm). (D) Epidermal thickness of mouse dorsal skin (*n* = 6). (E, F) Expression of TRIM27, p‐STAT3 and STAT3 in lesional skin of mice was detected by Western blot (*n* = 3). (G) Expression of IL‐1β, IL‐6 and IL‐8 in lesional skin of mice was detected by ELISA (*n* = 6). Data are expressed as mean ± SD. ***p < 0.001 compared with control. ^##^ *p* < 0.01, ^###^ *p* < 0.001 compared with IMQ.

To study the effect of m^6^A methylation on keratinocyte cytopathy in vivo, we applied imiquimod (IMQ) to shaved back skin of mice to produce psoriasis‐like inflammation,^35^ with one group treated by DAA and one group treated by a control vehicle cream. While the control group of mice developed obvious lesions on the skin, such symptoms were much lighter in mice treated with DAA (Figure 5A). The PASI scores showed significant reduction with DAA relative to the IMQ‐only group (*p* < 0.001; Figure 5B). Moreover, as shown by H&E staining, while mice with no treatment and with IMQ plus DAA treatment showed only minimal infiltration in the skin and a thinner epidermal spinous cell layer, and mice treated with IMQ but not DAA had massive inflammatory infiltrates and thickened epidermal spinous cell layer (*p* < 0.001; Figure 5C, D). Molecular analysis further showed that IMQ triggered the elevation of TRIM27 and p‐STAT3 levels (*p* < 0.001; Figure 5E, F) as well as the accumulation of proinflammatory cytokines IL‐1β, IL‐6 and IL‐8 (*p* < 0.001; Figure 5G) in the lesioned skin tissues, which were all alleviated by DAA treatment.

### 3.6 METTL14, TRIM27, STAT3 and p‐STAT3 expression are increased in patients with psoriasis

**FIGURE 6:**
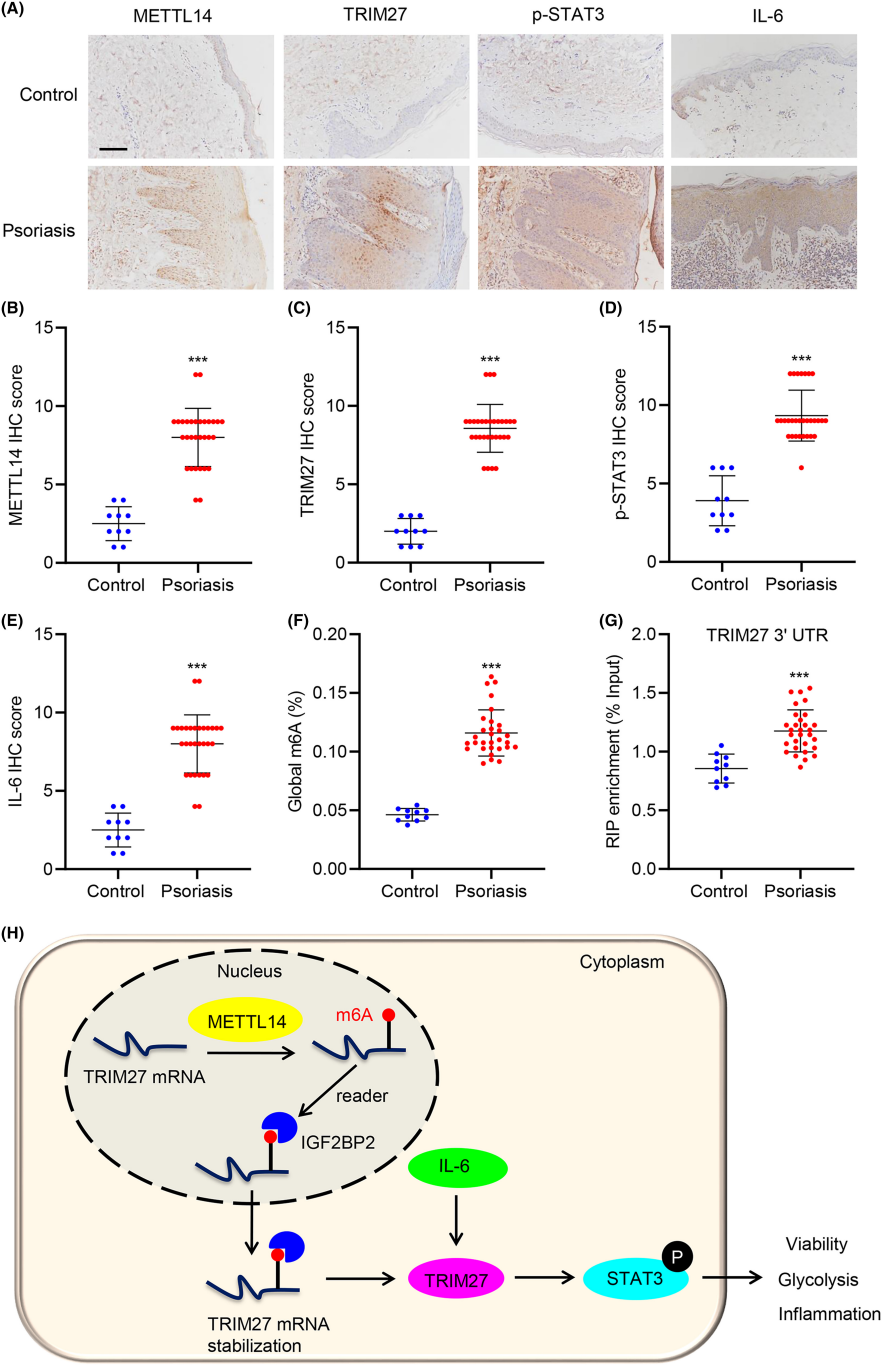
METTL14, TRIM27 and p‐STAT3 expression are increased in patients with psoriasis. (A–E) METTL14, TRIM27 and p‐STAT3 expression in psoriatic lesion tissues of patients with psoriasis (*n* = 30) and in healthy skin tissues of healthy volunteers (*n* = 10) as control were measured by IHC staining (Scale bar, 100 μm). (F) Global and (G) TRIM27 3′UTR m6A levels in psoriatic lesion tissues of patients with psoriasis (*n* = 30) and in healthy skin tissues of healthy volunteers (*n* = 10) as control. (H) Schematic representation of the relationship between IL‐6‐induced viability, glycolysis and inflammation and METTL14‐mediated m6A modification of TRIM27 in HaCaT cells. Data are expressed as mean ± SD. ***p < 0.001 compared with control.

Finally, we looked at whether our discoveries on HaCaT cells and on mice can be reflected in human pathology. Psoriasis is a representative disease with IL‐6 over‐expression^5^, ^6^ and cutaneous inflammation and epidermal proliferation.^36^ Therefore, lesioned skin tissues of clinical psoriatic patients were collected and processed by IHC staining. In comparison to skin tissues of healthy volunteers, these lesioned tissues showed elevated levels of METTL14, TRIM27, p‐STAT3, and IL‐6 expression (*p* < 0.001; Figure 6A–E). Moreover, the results showed a significant increase of global and TRIM27 3′UTR m6A levels in lesioned tissues of patients with psoriasis (*p* < 0.001; Figure 6F, G).

## 4 DISCUSSION

Skin forms one of the first barriers of human's defence against the environmental pathogens, and it is also a major site of immune and inflammatory responses. Skin diseases like psoriasis cause a huge burden in the mental health of individuals living with the condition, and patients with psoriasis often suffer from psoriatic arthritis and scleritis.^37^, ^38^ To better treat skin inflammation without suppressing the immune system requires in‐depth understanding of the inflammatory responses and related signalling pathways. In this work, we focused on a proinflammatory cytokine, IL‐6, and studied its signalling pathways in triggering keratinocyte cytopathy. Our results identified that IL‐6 activates METTL14 to trigger m^6^A methylation of TRIM27, which is recognized by IGF2BP2 to increase TRIM27 mRNA stability, leading to the activation of STAT3 and increased viability, glycolysis and inflammation in keratinocytes (Figure 6H). Scientifically, this work shed light on the molecular mechanisms of how IL‐6 regulates cell function. More importantly, it has the clinical meaning of pinpointing METTL14, TRIM27 and IGF2BP2 as three key regulators in the IL‐6/STAT3 signalling pathway for inducing increased viability, glycolysis and inflammation of keratinocytes.

The current treatment of inflammatory skin diseases often relies on the application of inhibitors of proinflammatory cytokines, which has the major drawback of inhibiting the immune system because proinflammatory cytokines play critical roles in immune responses by directing leukocytes towards sites of infection, thereby facilitating the elimination of pathogens. Psoriasis^5^, ^6^ and atopic dermatitis^39^, ^40^ are both characterized by elevated IL‐6 level. However, likewise, inhibiting the signalling of IL‐6 can lead to inability in defending infection.^39^, ^41^ In this context, the identification of intermediate signalling molecules METTL14, TRIM27 and IGF2BP2 in the IL‐6/STAT3 signalling pathway, and the unravelled activation mechanism of TRIM27, that is, m^6^A methylation, become clinically meaningful, because it allows the development of a new generation of drugs with them being the targets. Compared with drugs generally blocking the functionality of proinflammatory cytokines, targeting these intermediate signalling molecules can possibly selectively compromise the undesired effects of the cytokines without affecting others, thereby causing less effects to the immune system, which may solve the problem of immunosuppression.

IL‐6/STAT3 signalling pathway is an important signalling pathway in various types of cells. Based on the current understanding, IL‐6 can bind to IL‐6R (which can be both cell‐expressed and soluble) and induce the formation of a complex containing two copies of IL‐6, two copies of IL‐6R and two copies of gp130. In the complex, the cytoplasmic domain of gp130 would attract the docking of JAK, which in turn phosphorylates STAT3 and triggers its activation.^19^, ^42^ While the above description covers the backbone of the signalling pathway, many more regulators and mediators exist. For instance, STAT3 activation was found to activate a molecule named suppressor of cytokine signalling 3 (SOCS3), which inhibits the phosphorylation of STAT3 by JAK and therefore provides a negative feedback mechanism to the IL‐6/STAT3 signalling pathway.^43^ STAT3 activation is also essential for facilitating pro‐inflammatory factor releases including IL‐6, suggesting a positive feedback mechanism in IL‐6/STAT3 signalling pathway.^44^ In the present study, DAA, a methylation inhibitor, partly reduced IL‐6‐induced production of pro‐inflammatory factors such as IL‐1β, IL‐6 and IL‐8, which suggests that m6A modification may be partly involved in IL‐6/STAT3 singaling pathway mediated pro‐inflammatory factor releases. Although previous works have identified that TRIM27 serves as a scaffold protein in the IL‐6/STAT3 signalling pathway to stability the linkage of gp130, JAK1, and STAT3, thereby facilitating STAT3 phosphorylation,^18^, ^45^ it was never clear how TRIM27 is recruited to the complex and how it is activated upon IL‐6 stimulation. Here we for the first time discovered m^6^A methylation as the underlying mechanism of TRIM27 activation in this context, which is triggered by METTL14 and recognized by IGF2BP2 for activation. Overall, our work identified the METTL14/TRIM27/IGF2BP2 signalling axis as an amplifier of IL‐6 signalling in keratinocyte cytopathy. It is reasonable to suspect that the same signalling axis may also function in the context of other pathological conditions like cancer, which is worth future investigation. It would also be of both scientific and clinical importance to explore whether other facilitating/suppressing signalling axes exist for the IL‐6/STAT3 signalling pathway.

By forming a heterodimer with METTL3, METTL14 can act as a N6‐methyltransferase and methylates adenosine residues at the N (6) position of mRNAs to regulate a wide range of cell activities (e.g. genome repair) and suppression of various types of cancer (e.g. colorectal, liver, breast and skin tumorigenesis, etc.).^46^, ^47^ METTL14 was previously found to promote the m^6^A methylation of another member of the TRIM family, TRIM7, which is recognized by the m^6^A reader YTHDF2.^48^ Here we provided the first evidence showing that METTL14 can promote the m^6^A methylation of TRIM27 via IGF2BP2 acting as the reader, allowing METTL14 to directly contribute to IL‐6‐induced increased viability, glycolysis and inflammation in keratinocytes. This provides extra incentives for the development of METTL14 inhibitors, which can potentially be used as therapeutics for not only cancer,^47^ but also skin diseases like psoriasis and atopic dermatitis. Our study also have some limitations such as small sample sizes in vitro and in vivo experiments and in patients. Compared with HaCaT cells, a human immortalized keratinocyte, primary normal human epidermal keratinocytes should be further used for examing the role of METTL14‐mediated m6A modification of TRIM27 in regualting cell proliferation, glycolysis, and inflammation induced by IL‐6/STAT3 signalling pathway. Moreover, IL‐17 is well described in psoriasis and known to be involved in stabilizing K17 by modifying m6A.^49^ Therefore, its role in keratinocyte proliferation, glycolysis, and inflammation would be further examined.

## 5 CONCLUSION

We identified that in keratinocytes, IL‐6 activates METTL14 to trigger m^6^A methylation of TRIM27. This m^6^A methylation is recognized by IGF2BP2, which increases the stability of TRIM27 and facilitates its activation, eventually leading to the activation of STAT3 and increased viability, glycolysis and inflammation in keratinocytes. This is the first work that reports METTL14/TRIM27/IGF2BP2 signalling axis as an amplifier of IL‐6 signalling in keratinocyte cytopathy. Overall, this work expands our current understanding of the IL‐6/STAT3 signalling pathway, and emphasizes the potential of METTL14/TRIM27/IGF2BP2 signalling axis as a target for treating skin inflammation.

## AUTHOR CONTRIBUTIONS

**Yiran Chen:** Data curation (equal); formal analysis (equal); investigation (equal); methodology (equal). **Yanwei Xiang:** Formal analysis (equal); funding acquisition (equal); investigation (equal); methodology (equal). **Xiao Miao:** Investigation (equal); methodology (equal). **Le Kuai:** Formal analysis (equal); visualization (equal). **Xiaojie Ding:** Formal analysis (equal); visualization (equal). **Tian Ma:** Formal analysis (equal). **Bin Li:** Conceptualization (equal); project administration (equal); writing – review and editing (equal). **Bin Fan:** Conceptualization (equal); funding acquisition (equal); project administration (equal); supervision (equal); writing – original draft (equal).

## FUNDING INFORMATION

This study was supported by National Natural Science Foundation of China (82074302), Shanghai Municipal Commission of Economy and Information Technology, Shanghai Artificial Intelligence Innovation and Development Project‐Intelligent Dermatology Clinic Based on Modern TCM Diagnostic Technology (No. 2020‐RGZN‐02038), the National Key Reasearch and Development Program of China (No. 2018YFC1705305), and Evidence‐based dermotology base sponsored by State Administration of Traditional Chinese.

## CONFLICT OF INTEREST STATEMENT

No conflict of interest has been declared by the authors.

## Supporting information


FigureS1.

## Data Availability

The data that support the findings of this study are available from the corresponding author upon reasonable request.
