# Supplementary material for: METTL14 promotes IL‐6‐induced viability, glycolysis and inflammation in HaCaT cells via the m6A modification of TRIM27
Source: J Cell Mol Med. 2023 Dec 25;28(3):e18085. doi: 10.1111/jcmm.18085 (PMC10844716; doi:10.1111/jcmm.18085)
Supplement: Supplementary file 1 — FigureS1. [file JCMM-28-e18085-s001.docx]

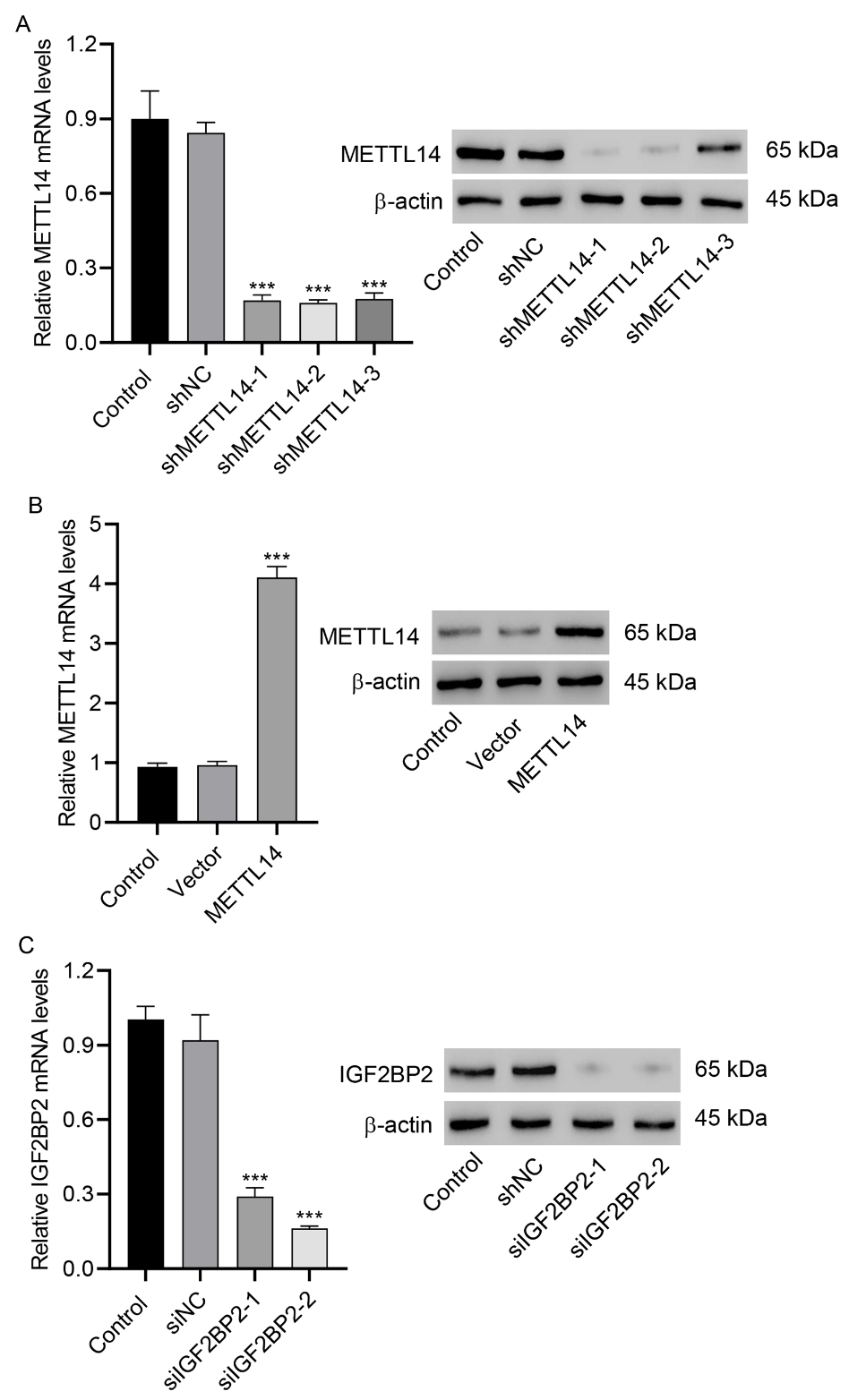


**Figure S1. METTL14 and IGF2BP2 expression levels in HaCaT cells.** (A, B) HaCaT cells were transduced with METTL14 shRNA or METTL14 expression vector and the METTL14 expression levels were used by Quantitative RT-PCR and Western blot. (C) HaCaT cells were transfected with IGF2BP2 siRNA and the IGF2BP2 expression levels were used by Quantitative RT-PCR and Western blot. Data are presented as mean ± SD from three independent experiments. ****P*<0.001 compared with shNC, siNC or vector.
